# Similarities and differences in the induction and regulation of the negative emotions fear and disgust: A functional near infrared spectroscopy study

**DOI:** 10.1111/sjop.12836

**Published:** 2022-05-30

**Authors:** Myrto Sklivanioti Greenfield, Yanlu Wang, Mussie Msghina

**Affiliations:** ^1^ Department of Clinical Neuroscience (CNS) Karolinska Institute Stockholm Sweden; ^2^ Department of Clinical Science Intervention, and Technology, Karolinska Institute Stockholm Sweden; ^3^ MR Physics, Medical Radiation Physics and Nuclear Medicine Karolinska University Hospital Stockholm Sweden; ^4^ Faculty of Medicine and Health Örebro University Örebro Sweden

**Keywords:** fNIRS, emotion, emotion induction, emotion regulation, fear, disgust, prefrontal cortex

## Abstract

Affective processing, including induction and regulation of emotion, activates neural networks, induces physiological responses, and generates subjective experience. Dysregulation of these processes can lead to maladaptive behavior and even psychiatric morbidity. Multimodal studies of emotion thus not only help elucidate the nature of emotion, but also contribute to important clinical insights. In the present study, we compared the induction (EI) and effortful regulation (ER) with reappraisal of fear and disgust in healthy subjects using functional near infrared spectroscopy (fNIRS) in conjunction with electrodermal activity (EDA). During EI, there was significant activation in medial prefrontal cortex (PFC) for fear and more widespread activation for disgust, with right lateral PFC significantly more active during disgust compared to fear. ER was equally effective for fear and disgust reducing subjective emotion rating by roughly 45%. Compared to baseline, there was no increased PFC activity for fear during ER, while for disgust lateral PFC was significantly more active. Significant differences between the two negative emotions were also observed in sympathetic nerve activity as reflected in EDA during EI, but not during ER. Lastly, compared to men, women had higher emotion rating for both fear and disgust without corresponding differences in EDA. In conclusion, in the present study we show that emotion induction was associated with differential activation in both PFC and sympathetic nerve activity for fear and disgust. These differences were however less prominent during emotion regulation. We discuss the potential interpretation of our results and their implications regarding our understanding of negative emotion processing.

## INTRODUCTION

Emotion is often operationalized as transient and prepotent response to salient external stimuli and/or internal mental representations (Ochsner & Gross, 2005; Phan, Fitzgerald, Nathan, Moore, Uhde & Tancer, 2005), involving changes across experiential, behavioral and physiological states (Gross & Barrett, 2011). Although a more detailed definition is a matter of debate (Adolphs, Mlodinow & Barrett, 2019; Clore & Ortony, 2013; Gross & Barrett, 2011; Mobbs, Adolphs, Fanselow *et al*., 2019), most experts accept that valence and arousal are important basic, domain‐general, orthogonal dimensions defining ‘core affect’ (Anderson & Adolphs, 2014; Barrett, Mesquita, Ochsner & Gross, 2007; Mobbs *et al*., 2019; Russell, 1980, 2003). Information with emotional content, especially of negative valence, is highly salient, receives priority in processing and its regulation is critical for optimal functioning (Banich, Mackiewicz, Depue, Whitmer, Miller & Heller, 2009; Fitzgerald, Kinney, Phan & Klumpp, 2018; Pessoa & Ungerleider, 2004; Troy, Wilhelm, Shallcross & Mauss, 2010).

Emotion can conceptually be studied from aspects of emotion generation, emotion perception and emotion regulation (Elliott, Zahn, Deakin & Anderson, 2011; Westgarth, Hogan, Neumann & Shum, 2021) and specifically, emotion regulation is of interest because of its clinical relevance, associated with a myriad of psychiatric diagnoses (Hu, Zhang, Wang, Mistry, Ran & Wang, 2014) and being the main target of various forms of psychological therapy methods (Bateman & Fonagy, 2010; Beck, 2005; Buhle, Silvers, Wager *et al*., 2014; Gross & Muñoz, 1995; Lynch, Trost, Salsman & Linehan, 2007; Renna, Quintero, Soffer *et al*., 2018; Rottenberg & Gross, 2007). Cognitive control of emotion comprises of a set of mental processes that exert top‐down regulation to optimize goal‐directed behavior in demanding situations, when automatic behavior is found to be suboptimal (Diamond, 2013; Espy, 2004; Miller & Cohen, 2001; Rahm, Liberg, Kristoffersen‐Wiberg, Aspelin & Msghina, 2014). Such cognitive control functions and the structures underlying them are the first to suffer during uncontrolled chronic stress, and the detrimental effects of the latter can be seen at the functional and structural level in prefrontal cortex and at the behavioral level in poorer performance and impaired ability to exercise self‐control (Arnsten, 1998; Banich *et al*., 2009; Diamond, 2013; Glausier & Lewis, 2018; Liston, McEwen & Casey, 2009; von Hecker & Meiser, 2005). The cognitive control of emotion, in line with cognitive control in general, involves different types of appraisal processes by continuously assessing the significance of stimuli to current goals and can be done, among others, at the cognitive level, namely reappraisal or at the behavioral level, namely suppression (Gross, 2015; Gyurak, Gross & Etkin, 2011; Nelson, Fitzgerald, Klumpp, Shankman & Phan, 2015; Ochsner & Gross, 2005; Phan *et al*., 2005; Phillips, Ladouceur & Drevets, 2008).

In this study, we focused on emotion induction and regulation of two emotions, fear and disgust. Although both are thought as being negatively valenced emotions, they are also considered distinct in many aspects. An important facet of emotion, for example, is its functional features (Adolphs, 2017; Adolphs, Mlodinow & Barrett, 2019; Barrett, 2012; Fanselow & Lester, 1988; Mobbs, Headley, Ding & Dayan, 2020). Identification of physical threat is thought to activate survival mechanisms and potentially contribute to the emergence of a conscious experience of fear, which would then guide choice of a suitable behavior from a wide repertoire of possible actions (LeDoux, 2014; Mobbs *et al*., 2020). Disgust, on the other hand, is thought to have evolved from a phylogenetically primitive sensation of distaste (Calder, Lawrence & Young, 2001; Rozin & Fallon, 1987) and to be associated with disease threat (Anderson & Rutherford, 2012). Furthermore, fear and disgust are considered to have differences in expression (Ekman, 1992), physiology (Ekman, Levenson & Friesen, 1983; Lang, Greenwald, Bradley & Hamm, 1993; Woody & Teachman, 2000), cognitive appraisal (Cisler, Olatunji & Lohr, 2009), genetic influence (Gray, Young, Barker, Curtis & Gibson, 1997) and neural activity (Phan, Wager, Taylor & Liberzon, 2004; Phillips, Young, Senior *et al*., 1997). Specific emotions such as sadness, fear and disgust are assumed to make up core symptoms of affective and anxiety disorders, including certain aspects of obsessive–compulsive disorder (OCD) (APA, 2013; Berle, Starcevic, Brakoulias *et al*., 2012; Cisler *et al*., 2009; Etkin & Wager, 2007; Woody & Teachman, 2000); which is why, we argue, studying specific categories of emotion could lead to important clinical insights. Emotion regulation is central in almost all psychotherapeutic interventions targeting affective and non‐affective disorders, both traditional therapies such as cognitive behavioral therapy (CBT) and novel ones such as emotion regulation therapy (ERT) (Beck, 2005; Renna, Quintero, Fresco & Mennin, 2017).

Studies of emotion induction and emotion regulation paradigms in healthy subjects, often in conjunction with neuroimaging, have outlined important elements of emotion processing (Buhle *et al*., 2014; Ochsner, Bunge, Gross & Gabrieli, 2002; Phan *et al*., 2005, 2004). For example, although there is considerable overlap, studies with functional neuroimaging have shown that disgust preferentially activates insula, while fear entails more often amygdala activation (Cisler *et al*., 2009; Phan, Wager, Taylor & Liberzon, 2002). There is also evidence suggesting that emotion regulation strategies for fear and disgust may be variably effective and that disgust may be especially difficult to modulate cognitively (Rozin & Fallon, 1987; Sheppes & Meiran, 2007), possibly requiring behavioral suppression, explicitly intended or automatic. Interestingly, there is data that indicates that reappraisal may preferentially activate middle and superior frontal gyri, while suppression is associated with activation of inferior frontal gyrus, which is important for inhibition of motor responses associated with emotional reactivity (Frank, Dewitt, Hudgens‐Haney *et al*., 2014).

Functional near infrared spectroscopy (fNIRS), with a superior ecological validity that allows more naturalistic experimental settings compared to other imaging methods and easy accessibility of the prefrontal cortex, is increasingly being used to probe cognitive and affective processes in cortical areas (Bendall, Eachus & Thompson, 2016; Crum, 2020; Doi, Nishitani & Shinohara, 2013; Westgarth *et al*., 2021). fNIRS allows the determination of relative changes in the concentration of oxygenated (oxy‐Hb) and deoxygenated hemoglobin (deoxy‐Hb), while simultaneous functional magnetic resonance imaging (fMRI) and fNIRS recordings have shown that the blood oxygen level‐dependent (BOLD) and fNIRS signals are strongly correlated to each other (Cui, Bray, Bryant, Glover & Reiss, 2011; Strangman, Culver, Thompson & Boas, 2002). Previous fNIRS studies have shown a right lateralized activation of prefrontal cortex for negative and left lateralized activation for positive emotions (Balconi, Grippa & Vanutelli, 2015b; Everhart & Harrison, 2000; Tanida, Katsuyama & Sakatani, 2007). Hoshi, Huang, Kohri *et al*. (2011) using event‐related fNIRS showed that negative emotions increased oxy‐Hb in bilateral ventrolateral prefrontal cortex (PFC), and positive emotions decreased it in the left dorsolateral PFC. Tupak, Dresler, Guhn *et al*. (2014), also using fNIRS, showed that implicit or automatic emotion regulation activated ventrolateral PFC areas, as Hariri, Mattay, Tessitore, Fera and Weinberger (2003) had previously shown using fMRI. Herrmann, Ehlis and Fallgatter (2003) using emotion induction tasks with and without requirement for self‐monitoring showed that although emotion rating did not differ between the two tasks, the left PFC was activated during the emotion induction task requiring self‐monitoring, but not during the task that did not require self‐monitoring.

Differences in the peripheral physiology of fear and disgust have also been discerned in numerous studies (Ekman *et al*., 1983; Lang *et al*., 1993; Woody & Teachman, 2000). The sympathetic branch of the autonomic nervous system regulates the activity of the eccrine sweat glands of the skin, affecting skin conductance and making the later one of the most frequently used measures of sympathetic activity. Moreover, electrodermal activity (EDA) has been thought as a potential peripheral biomarker of central affective processes and has been shown, among other things, to reflect limbic and ventral prefrontal activations (Furmark, Fischer, Wik, Larsson & Fredrikson, 1997; Lang, Davis & Ohman, 2000). Previous fNIRS studies have reported correlation between PFC activation and sympathetic activity in response to mental tasks (Balconi *et al*., 2015b; Tanida *et al*., 2007) and other studies have shown that EDA responses differ depending on the kind of the underlying affective process (Balconi, Brambilla & Falbo, 2009; Balconi *et al*., 2015b; Cuthbert, Schupp, Bradley, Birbaumer & Lang, 2000; Tupak *et al*., 2014). EDA is thus considered an important complementary method of studying the peripheral autonomic activity in parallel with that of central nervous system activity.

A prevailing notion has been that women show higher sensitivity and reactivity to negatively valenced stimuli compared to men (Bradley, Codispoti, Sabatinelli & Lang, 2001; Deng, Chang, Yang, Huo & Zhou, 2016; Kring & Gordon, 1998), and that processes related to physical threat (conceptualized as fear) and disease and contamination (conceptualized as disgust) show gender differences (Pearson, Lightman & Evans, 2009; Swain, Lorberbaum, Kose & Strathearn, 2007). Previous fMRI and fNIRS studies have indeed shown gender differences in emotion processing, with women showing greater neural activation for negative emotions than men (Bradley *et al*., 2001; Stevens & Hamann, 2012; Yang *et al*., 2007)

### The present study

To our knowledge, no previous fNIRS study has investigated similarities and differences in the induction and regulation of different negative emotions. In the present study, we compared the induction and regulation of fear and disgust, using fNIRS in conjunction with EDA in healthy subjects to investigate whether prefrontal and sympathetic nerve activity differed during the induction and regulation of these two negative emotions. We first hypothesized that prefrontal cortex (PFC) and sympathetic nerve activity would differ between emotion induction and emotion regulation, with emotion regulation showing less PFC and sympathetic nerve activation. Second, we hypothesized that fear and disgust would differentially activate PFC and sympathetic nervous system during emotion induction, a difference we expected to be less prominent during emotion regulation due to attenuation of emotion intensity. Based on previous literature, we predicted an increased sympathetic nerve activity during induction of fear and lower than baseline sympathetic nerve activity during induction of disgust. Lastly, we sought to investigate the role of gender in this, given that previous studies had shown gender differences in affective processes.

## METHODS

### Participants

Subjects were recruited from a non‐clinical population by advertisement in Psychiatry Southwest and Karolinska University Hospital, Huddinge. The sample size (*n* = 45; mean age 31.9 ± *SD* 9.3 years; 56% females) was calculated after performing a power analysis based on a pilot study (Suresh & Chandrashekara, 2012), see also *Supplementary material*. Prior to the start of the experiments, subjects were given an overview of the general scope of the study and the outline of the experimental procedure. All subjects met the following inclusion criteria: able and willing to provide written informed consent, 18 years of age or older at the time of recruitment, free of any psychiatric, neurological and addiction disorders as well as any current drug use including psychoactive medication. All subjects were asked to abstain from alcohol consumption at least one day prior to the trial and were instructed to continue their usual consumption of coffee and nicotine and keep it the same level prior to each part of the testing.

### Ethics

The study was approved by the Stockholm County’s ethics committee (Dnr 2013/722‐32 and 2014/ 436‐32). All subjects were given verbal and written information and gave written informed consent through their signature prior to the start of the experiment, in accordance with the Declaration of Helsinki.

### Study design

#### Experimental design

The experimental setup included a counterbalanced block design with a randomized order of sequence of the two tasks (FEAR and DISGUST), examining the induction and regulation of the two negative emotions.

**Fig. 1:**
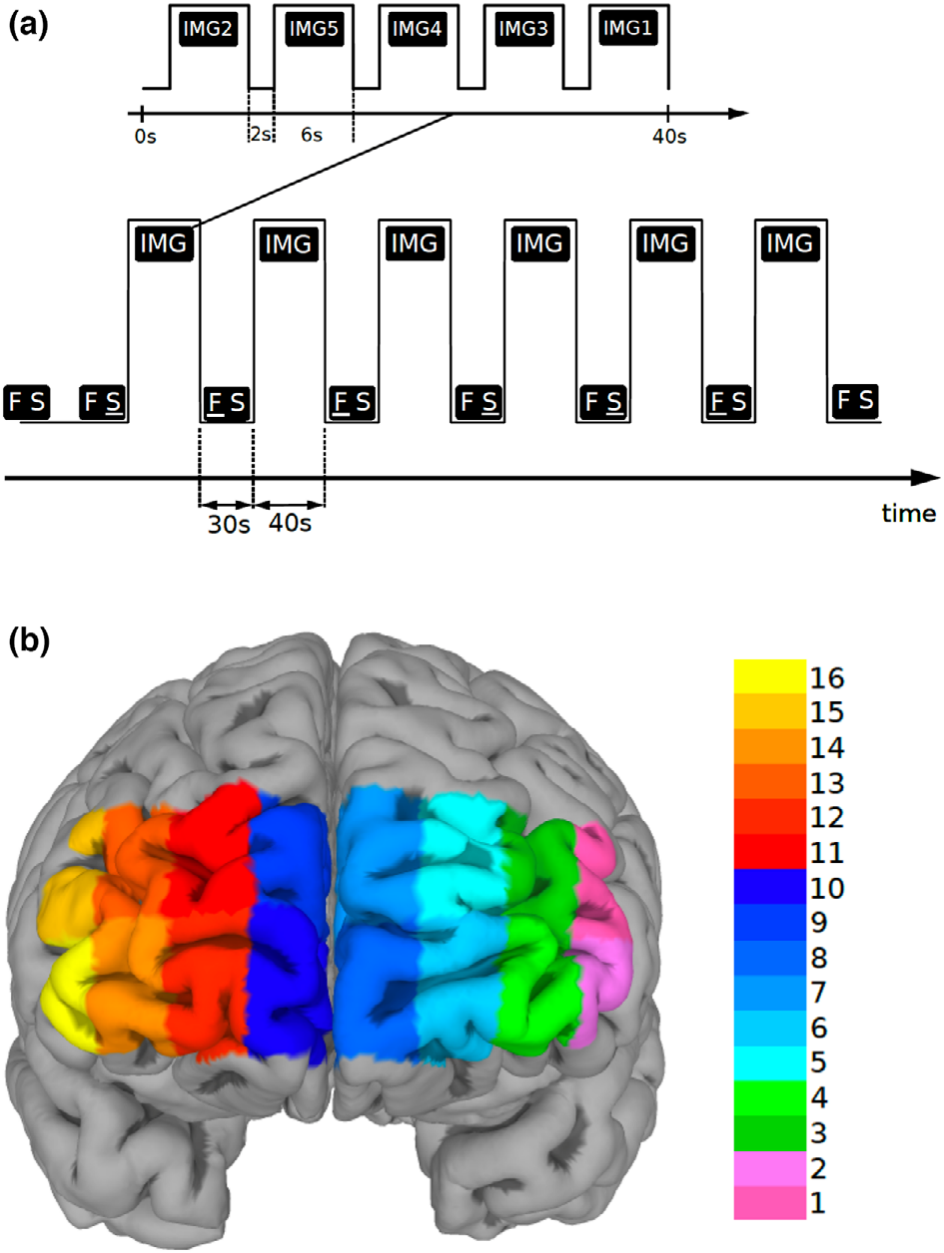
(a) Paradigm timing for each task (fear and disgust). Each task included six blocks of stimuli (IMG), each block was 40‐s long and was preceded by a 30‐s long period of REST (FS). In each block, five different visual emotive stimuli (pictures IMG1‐5) were presented on the screen, each for six seconds, followed by a two‐seconds long interval to separate them from each other. Immediately after viewing the pictures, the subjects were asked to score emotion intensity for every image in a scale ranging from 1 to 9 during emotion induction (EI) and emotion regulation (ER). (b) Corresponding fNIRS channel locations on the PFC. See Methods for details. [Colour figure can be viewed at wileyonlinelibrary.com]

Each task included six blocks of stimuli, each block was 40 s long and was preceded by a 30 s long period of REST. In each block, five different stimuli, that is, pictures representative for respective emotion, were presented, each for six seconds, followed by a two‐seconds long interval to separate them from each other (see Fig. 1a).

The six blocks were randomly assigned in a counterbalanced way to one of the two conditions, either Emotion Induction or Emotion Regulation, resulting in each of these two conditions being repeated three times and allowing the functional response to be disentangled from the physiological confounds. During REST the screen displayed two letters “F S” on a white background, the letter F underlined (F S) denoting the ensuing pictures as an Emotion Induction block (EI), whereas when the letter S was underlined (F S) the succeeding block was denoted as an Emotion Regulation block (ER). More specifically, study subjects were told that “when the letter ‘F’ is underlined, you are to simply look at the pictures. However, when the letter ‘S’ is underlined, you will try to reduce the intensity of emotion the pictures generate by reevaluating or reinterpreting the significance of the picture.” In addition, they were given examples of reappraisal and were instructed not to use other emotion regulation methods, such as suppression of emotion. In order to reduce carry‐on and anticipatory effects, the order of conditions as well as the order of the stimuli within each block were randomized. The tasks were implemented in E‐Prime (version 2.1, http://www.pstnet.com/eprime.cfm).

Primary and secondary outcomes were defined for the behavioral, fNIRS and EDA measures. Regarding behavioral data, we used Emotion Induction score and Emotion Regulation index as primary outcomes. The fNIRS analysis was based on region‐wise analysis of left (LPFC), right (RPFC) and medial prefrontal cortex (MPFC), with secondary post‐hoc channel‐wise analysis. Lastly, electrodermal response (EDR) frequency was defined as the primary outcome for electrodermal activity (EDA), and EDR and electrodermal level (EDL) amplitudes as secondary outcomes subjected to exploratory analyses.

#### Emotion induction and emotion regulation tasks

The International Affective Picture System (IAPS, Lang *et al*., 2008) was used as a source of the standardized pictures for the tasks. Threat and physical harm related images were selected for fear and disease and contamination related images for disgust. Additionally, the images were explicitly labelled in each block as representing fear or disgust, respectively (see *Supplementary material* for details and id number of the selected images for each emotion). As described above, the subjects were instructed to either passively view the ensuing pictures (Emotion Induction, EI) or to actively down‐regulate the emotion using reappraisal (Emotion Regulation, ER). To ascertain that test subjects conformed to the instructions given to them, at least as far as they could tell, they were interviewed immediately after the end of the experiment as to the specific strategy they used, which was documented verbatim. To limit the effect of confounds, such as temperature and humidity, that can affect measurements, we performed the experiment in a closed room with stable and regulated temperature and humidity and measurement was started several minutes after the subjects had stayed in the room connected to the fNIRS device and reported feeling relaxed and comfortable.

#### Data analysis

We used a block‐design to assess differences between tasks and conditions. A set of multilevel mixed‐effects linear regression models (fixed effects: EMOTION (fear and disgust) × CONDITION (emotion induction and emotion regulation), random effects: intercepts for subjects due to repeated measures; method of estimation: maximum likelihood) were applied to the dependent measures of primary outcomes (behavioral measures, fNIRS and EDA data) and the Benjamini‐Hochberg method used to control for multiple testing (raw *p*‐values are reported). Two‐tailed *t*‐tests, with the probability of rejecting the null hypothesis set at *p* < 0.05 (adjusted according to the Bonferroni correction method), were subsequently performed to explore significant contrasts in comparison to rest or baseline conditions for fNIRS and EDA. Normality was tested and non‐parametric tests were performed where relevant. We also performed post‐hoc exploratory analyses of the behavioral and EDA data for the purpose of examination of the effects of gender on our data as abundant previous literature suggests it has a major role (Olatunji, Taylor & Zald, 2019; Skolnick, 2013; Stevens & Hamann, 2012). However, our study did not have sufficient power to expand this post‐hoc analysis to include the fNIRS measures. Lastly, we performed an exploratory post‐hoc, channel‐wise analysis for the fNIRS data where we applied nominal testing (Moyé, 2008), see also *Supplementary material*. All statistical analysis was performed using Stata 14 software (StataCorp., College Station, TX, USA).

### Data acquisition

#### Behavior measures of subjective emotion experience

Immediately after viewing the pictures, the subjects were asked to score emotion intensity for every image in a scale ranging from 1 to 9 during emotion induction (EI) and emotion regulation (ER), where 1 represented lowest and 9 highest level of emotion intensity (participants were instructed to “score the intensity of the experienced emotion in response to the image in a scale from 1–9, 1 representing the least and 9 the most intense feeling”). The mean score for the images was calculated to represent subjective emotion rating for each condition (EI and ER). Emotion regulation index was calculated based on the subjective rating during emotion induction (EI) and emotion regulation (ER) using the following formula:

$$\text{Emotion Regulation Index}=\left(\mathrm{EI}-\mathrm{ER}\right)/\mathrm{EI}$$

#### Functional near‐infrared spectroscopy (fNIRS) recordings

A continuous wave fNIRS device consisting of a flexible headband holding light sources and detectors (fixed distributions), and a fNIR100 data acquisition box with a sampling rate of 2 Hz connected to a personal computer via an MP150 data acquisition and analysis system (Biopac Systems Inc. Goleta, CA, USA, JOR AB, Knivsta, Sweden) was used to measure the relative changes in the concentration of oxy‐hemoglobin (Δoxy‐Hb). The headband was placed on the forehead of the participant and the sensor consisted of four infrared light sources emitting at two different wavelengths (730 and 850 nm) and ten detectors separated by a distance of 2.5 cm, giving a total of 16 channels for recording different parts of the prefrontal cortical mantle (mainly BA 9, 10, 45, 46 [Ayaz, Onaral, Izzetoglu, Shewokis, McKendrick, & Parasuraman, 2013]), see Fig. 1b). Electrode placement was done according to the protocol recommended by Biopac Systems Inc. and as described by Ayaz, Shewokis, Curtin, Izzetoglu, Izzetoglu and Onaral (2011) and visualized by Ayaz et al., (2013) in Fig. 7. Participants were asked to lift their hair off the forehead before sensor placement, the sensor strip was placed just above the eyebrows and the center of the sensor was matched with the vertical axis of symmetry that passes through the nose. Data acquisition was performed using the Cognitive Optical Brain Imaging Studio software (fNIR Devices LLC, Potomac, MD, USA) and a second personal computer was connected to the system via a COM cable to synchronize the E‐Prime data set with the fNIRS and electrodermal activity (EDA) data sets using Acqknowledge software version 4.2 (Biopac Systems Inc. Goleta, CA, USA, JOR AB, Knivsta, Sweden). Raw light intensity data was automatically converted to levels of oxygenated (HbO) and deoxygenated hemoglobin (HbR) by COBI software utilizing the modified Beer–Lambert Law.

##### Preprocessing and statistical analysis

For the fNIRS data we used “NIRS‐SPM toolbox” (Tak, Uga, Flandin, Dan & Penny, 2016) that utilizes the SPM12 package (Wellcome Department of Cognitive Neurology, London, UK) and runs under MATLAB (MATLAB_R2019b, Mathworks, Natick, MA). In the present study, we chose to report Oxy‐Hb, because this chromophore measures more reliably cerebral blood flow and task‐related activation (Bendall *et al*., 2016; Hock, Müller‐Spahn, Schuh‐Hofer, Hofmann, Dirnagl & Villringer, 1995; Kameyama, Fukuda, Yamagishi *et al*., 2006; Tanida *et al*., 2007; Tanida, Sakatani, Takano & Tagai, 2004). Indeed, many studies use Oxy‐Hb (Burns, Barnes, Katzman, Ames, Falk & Lieberman, 2018; Causse, Chua & Rémy, 2019; Hoshi *et al*., 2011; Ren, Lu, Liu *et al*., 2017; Tanida *et al*., 2007), not only because it is a better indicator of task‐related activity (Hoshi *et al*., 2011), but also because it has better signal‐noise ratio (Hoge, Franceschini, Covolan, Huppert, Mandeville & Boas, 2005; Strangman *et al*., 2002), and correlates well to fMRI BOLD signal (Cui *et al*., 2011). On the other hand, deoxy‐Hb has better spatial resolution (Franceschini, Toronov, Filiaci, Gratton & Fantini, 2000), but is known to suffer from low signal‐noise ratio limiting its usability (Balconi, Grippa & Vanutelli, 2015a; Bulgarelli, Blasi, Arridge *et al*., 2018; Tam & Zouridakis, 2015). Physiological noise (i.e., artifacts from respiration and cardiac pulsation) was removed using two band‐stop filters (0.12–0.25 and 0.7–2.0 Hz). Since our paradigm did not involve movement as part of the experiment, and our subjects mostly complied with the instruction to sit still during the experiments, visual inspection of our data showed minimal motion artifacts and comparison of our data with and without motion correction (Scholkmann, Spichtig, Muehlemann & Wolf, 2010)) showed no substantial differences, and we therefore did not correct for movement artefacts. Less than 5% of the channels (without specific channels being overrepresented) in all trials were excluded because of technical quality problems. For detrending and reducing low‐frequency confounders, a high‐pass filter based on a discrete cosine transform set with the cut‐off period set to 128 s was utilized. Autocorrelations in the time series due to hemoglobin changes were corrected using the pre‐whitening method from the NIRS‐SPM toolbox (Purdon & Weisskoff, 1998).

Using a generalized linear model (GLM), the data from each channel was separately fitted to the ideal responses modelled through the onset timings with the hemodynamic response function consisting of the canonical HRF and its temporal and dispersion derivatives. Two *t*‐contrasts were calculated: (1) Emotion Induction contra Rest [EI – REST]; and (2) Emotion Regulation contra Rest [ER – REST]. As a result, channel‐specific beta coefficients were generated which were used for further statistical analyses. The data were averaged over left (LPFC, Channels 1–6), medial (MPFC, Channels 7–10) and right (RPFC, Channels 11–16) prefrontal regions to increase signal‐to‐noise ratio but we also report a post‐hoc, exploratory, channel‐wise analysis with uncorrected p‐values (Moyé, 2008), see *Supplementary material*. Outlier correction was performed by replacing outliers with the Q1–1.5 IQR and Q3 + 1.5 IQR rather than outright removing them, as a more conservative approach.

#### Electrodermal activity (EDA)

To record electrodermal activity (EDA), two non‐polarizable Ag‐AgCl electrodes (EL 507, JOR AB, Knivsta, Sweden) were placed on the middle phalanges of digits 2 and 3 of the left hand (exosomatic recordings using a direct current) connected to a GSR100C amplifier of the Biopac MP150 system, and data acquisition performed using Acqknowledge software version 4.2 (Biopac Systems Inc. Goleta, CA, USA, JOR AB, Knivsta, Sweden). Amplifier gain was set at 10 μmho/V, low‐pass filter at 1 Hz and high‐pass filter at 0.05 Hz. After data acquisition a low‐pass filter (5th‐order low‐pass Butterworth filter with cut‐off frequency at 1 Hz) and a median smoothing (smoothing window equal to the sample frequency 8 Hz) were applied to the raw EDA signals to remove high‐frequency noise. The preprocessed signal was then decomposed into three components: tonic signal, phasic signal and white Gaussian noise using a convex optimization approach (Greco, Valenza, Lanata, Scilingo & Citi, 2016). Lastly three variables were extracted using Matlab EDA Toolbox (https://github.com/mateusjoffily/EDA/wiki): (1) the frequency of the phasic electrodermal response (EDR); (2) the magnitude of EDR; and (3) the mean amplitude of the tonic electrodermal activity level (EDL), for REST (non‐specific EDR) as well as Emotion Induction (EI) and Emotion Regulation (ER) periods (stimulus evoked EDR). The latency window for stimulus evoked EDR onset was set to 1–3 s after stimulus onset.

## RESULTS

### Behavioral data

**Fig. 2:**
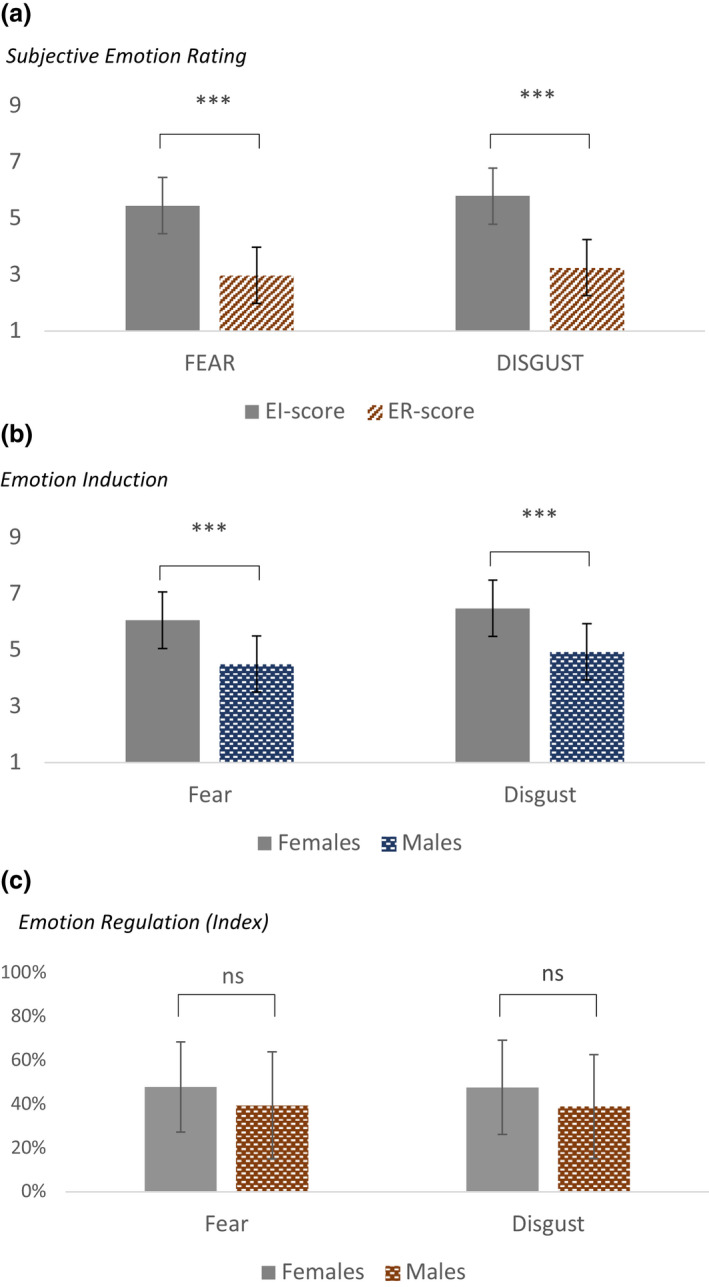
(a) Shows reduction of subjective emotion rating during Emotion Induction (EI) and Emotion Regulation (ER). Emotion regulation with reappraisal reduced subjective emotion rating by roughly 45% for both FEAR and DISGUST (mean ± *SD*). (b) Gender differences in emotion rating during EI. Female subjects had roughly 26% and 24% higher emotion rating for FEAR and DISGUST, respectively, than male subjects. (c) Gender differences in emotion rating during ER. Female subjects had roughly 17% and 19% higher emotion rating for FEAR and DISGUST, respectively, compared to male subjects, although these were not statistically significant (shown are mean ± *SD*). [Colour figure can be viewed at wileyonlinelibrary.com]

For both FEAR (contrast −2.48, SE 0.22, 95% CI [−2.9, −2.05], p < 0.001) and DISGUST (contrast −2.55, SE 0.22, 95% CI [−2.98, −2.12], p < 0.001), subjects rated the emotion higher when they simply attended to the pictures (EI), compared to when they actively tried to downregulate them using reappraisal (ER). For FEAR, ER was more efficient the higher the emotion rating was (*p* = 0.033), but we saw no such variation in the efficiency of ER with emotion rating for DISGUST (*p* = 0.3). There was a mean 44.0% reduction in emotion rating (ER index) for FEAR (95% CI [37.2%, 50.8%], *p* < 0.001) and 43.1% for DISGUST (95% CI [36.4%, 49.8%], *p* < 0.001) during ER compared to EI, with no significant difference between the two negative emotions (mean difference − 1%, 95% CI [−2.5%, 0.4%], *p* = 0.593) (Fig. 2a).

The pictures chosen for FEAR and DISGUST from the IAPS were relatively well calibrated and generated comparable emotion intensity, and the fixed effect interaction between EMOTION × CONDITION revealed no significant differences between the two emotions during EI (contrast 0.38, SE 0.22, 95% CI [−0.05, 0.8], p = 0.08).

Compared to males, female subjects had higher emotion rating during EI for both FEAR (mean difference 1.56 in a scale 1–9, 95% CI [0.7, 2.39], p < 0.001) and DISGUST (mean difference 1.54, 95% CI [0.67, 2.42], *p* < 0.001) (Fig. 2b). Female subjects also had higher ER efficiency compared to males, but this was not statistically significant (mean difference for ER index FEAR 17.4%, *p* = 0.22, and for DISGUST 18.6%, *p* = 0.21) (Fig. 2c).

### Functional near‐infrared spectroscopy recordings (fNIRS)

**Fig. 3:**
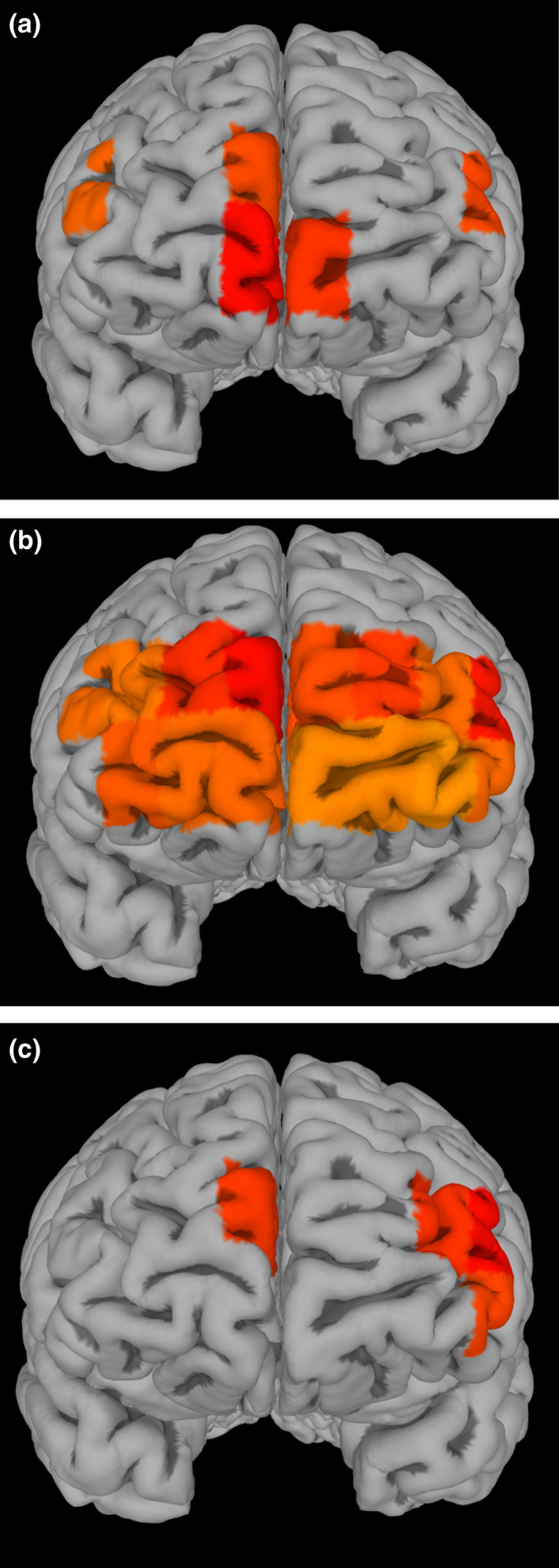
Shows fNIRS significant activations (Δoxy‐Hb) based on channel‐wise analysis for FEAR (a) and DISGUST (b); differences in Δoxy‐Hb levels during Emotion Induction (EI) [EI – REST]; significant activations were seen in medial and lateral PFC for FEAR and in more widespread areas for DISGUST. (c) Differences in Δoxy‐Hb levels during ER [ER – REST] during DISGUST; significant activations were seen in left lateral PFC. For FEAR there was a tendency for increased activity in ventromedial PFC during ER that did not reach statistical significance (not shown here, see *Supplementary material*). [Colour figure can be viewed at wileyonlinelibrary.com]

The fixed effects of the interaction between EMOTION × CONDITION revealed statistically significant differences between ER [ER – REST] and EI [EI – REST] for DISGUST (MPFC contrast −0.41, SE 0.18, 95% CI [−0.78, −0.04], *p* = 0.02 and RPFC contrast −0.34, SE 0.17, 95% CI [−0.67, −0.01], *p* =0 .04) but not for FEAR. When looking at activation patterns separately during ER [ER – REST] and EI [EI – REST], in both FEAR and DISGUST, there were significantly greater activations during EI compared to baseline conditions (REST). For FEAR in EI, significant activations were seen in MPFC [*t*(41) =2.75, p = 0.026] but no significant activations in ER (see also channel‐wise activations in Fig. 3a and *Supplementary material*). For DISGUST in EI, greater than REST prefrontal activation was more widespread covering almost all fNIRS channels [LPFC t (42) = 3.33, p = 0.005; RPFC *t*(42) = 3.29, p = 0.006; MPFC *t*(42) = 3.9, p = 0.001], whereas for ER, significant activations were seen only in left lateral PFC [LPFC *t*(42) = 2.7, p = 0.029], see also Fig. 3b, 3c and *Supplementary material*.

The fixed effects of the interaction between EMOTION x CONDITION, was significant for the contrast between the two emotions (RPFC contrast 0.33, SE 0.17, 95% CI [0.01, 0.67], *p* = 0.04) during the EI condition, revealing a stronger activation for DISGUST in this area of PFC. There was no significant difference between the two emotions during ER. We found no statistically significant results for deoxy‐Hb (see *Supplementary material*, section 3.2.1.2).

### Electrodermal activity (EDA)

Regarding the frequency of phasic electrodermal response (EDR), the fixed effects of the interaction between EMOTION × CONDITION showed statistically significant differences between ER [ER – REST] and EI [EI – REST] for both FEAR (contrast 0.09, SE 0.01, 95% CI [0.07, 0.1], *p* < 0.001) and DISGUST (contrast 0.11, SE 0.01, 95% CI [0.098, 0.12], *p* < 0.001).

There were no significant differences in EDA for FEAR during EI compared to resting conditions. For DISGUST, however, there was significantly lower EDR frequency [*t*(42) = 4.38, *p* < 0.001] and magnitude [*t*(42) = 2.59, *p* = 0.04] during EI compared to resting conditions.

**Fig. 4:**
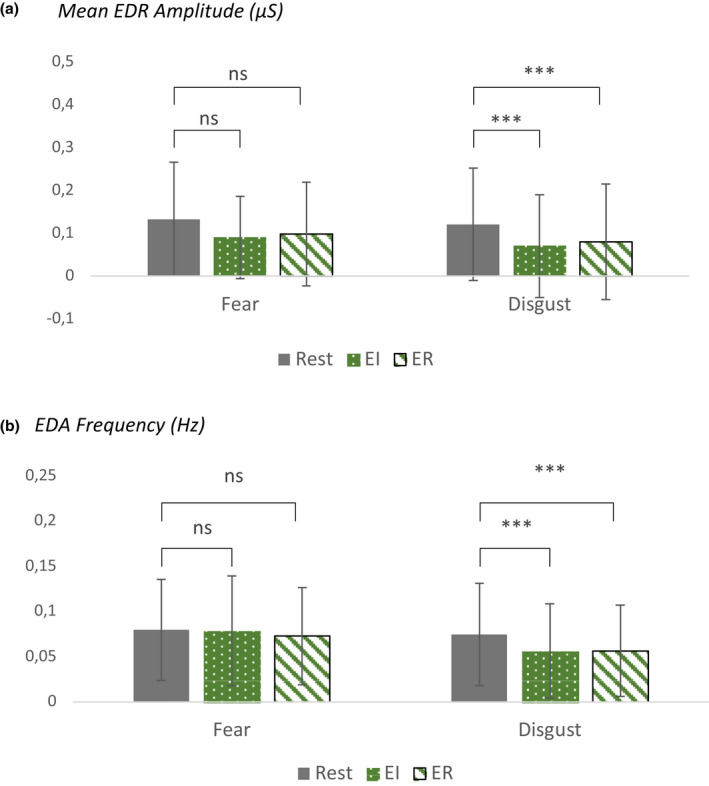
Shows measures of phasic components of electrodermal activity, mean phasic electrodermal response (EDR) amplitude (a) and EDR frequency (b). During EI, we measured increased sympathetic arousal during FEAR, but decreased sympathetic activity during DISGUST. During ER, we observed a reduction of sympathetic activity as reflected in changes in EDA both for FEAR and DISGUST. The two emotions differed significantly only during EI in terms of EDR frequency (shown are mean ± *SD*). [Colour figure can be viewed at wileyonlinelibrary.com]

During ER, EDR magnitude was lower for FEAR compared to resting conditions [*t*(38) = 2.78, *p* = 0.03]. For DISGUST, both EDR frequency [*t* (42) = 4.26, *p* < 0.001] and amplitude [*t*(42) = 2.72, *p* = 0.028] were lower during ER compared to baseline (see Fig. 4a and b).

Comparing the two emotions under EI conditions [contrast EI ‐ REST), we found that FEAR had significantly higher EDR frequency compared to DIGUST (contrast 0.015, SE 0.01, 95% CI [0.028, 0.002], p = 0.021). There was no difference between the two emotions under ER conditions (*p* = 0.19).

We found no significant differences in EDA between males and females, neither for EDR frequency and magnitude nor tonic EDA levels.

## DISCUSSION

In the present study, we compared the induction and regulation of two negative emotions, fear and disgust, using functional near infra‐red spectroscopy in conjunction with electrodermal activity. Effortful emotion regulation is conceptualized as the ability to exert voluntary control over emotional experience and the ensuing behavior (Gross, 1998; Ochsner & Gross, 2005; Phillips, Drevets, Rauch & Lane, 2003), and cognitive control using reappraisal has been shown to be an effective strategy in increasing or decreasing subjective rating of emotions (Gross, 2013; Ochsner *et al*., 2002). In parallel with effortful cognitive control, emotion regulation occurs also implicitly and automatically by constantly evaluating stimuli and affective experiences (Braunstein, Gross & Ochsner, 2017; Fitzgerald *et al*., 2018). In this study, we employed a similar paradigm as that used by Ochsner *et al*. 2002 to evaluate similarities and differences in the induction and regulation of the negative emotions fear and disgust and expected that effortful emotion regulation would substantially reduce subjective emotion rating, blunt sympathetic responses and alter activity in pertinent areas of the prefrontal cortex (Ochsner *et al*., 2002, 2004). Effortful emotion regulation with reappraisal is a robust paradigm that allows conclusions pertaining to emotion regulation to be drawn, both in the short term (Ochsner *et al*., 2002, 2004) and long term as a result of psychotherapeutic interventions (Beauregard, 2014). In our study, we compared two active conditions (emotion induction and regulation) on two basic emotions (fear and disgust). The lack of a neutral condition with images without emotive stimuli being a limitation of this study somewhat restricts the conclusions that can be drawn.

### Behavioral data

In line with previous studies, we found that effortful emotion regulation with reappraisal reduced subjective emotion rating by roughly 45% for both fear and disgust, with no significant difference in this between the two negative emotions when compared to each other. An interesting difference between the two negative emotions that we saw was that emotion regulation for fear was more efficient the higher the emotion rating, while for disgust there was no such variation in the efficiency of emotion regulation with emotion rating. This may indicate difference in capacity or flexibility between the emotion regulation processes activated by fear and disgust, as it has indeed been reported that disgust may be more difficult to modulate cognitively (Rozin & Fallon, 1987; Sheppes & Meiran, 2007). Disgust would also be expected to elicited emetic reactions, that may turn out to be less amenable to cognitive control. Also, anxiety disorders, such as GAD, that are assumed to be related to fear and others, like OCD, assumed to be related to disgust (Bhikram, Abi‐Jaoude & Sandor, 2017) appear to respond differently to psychological treatment, with therapies mediating cognitive restructuring recommended for GAD and those focusing on exposure and response prevention for OCD (Cuijpers, Sijbrandij, Koole, Huibers, Berking & Andersson, 2014; Robbins, Vaghi & Banca, 2019). These discrepancies could reflect differences in the nature of these two emotions, and to the differences we observed in the efficiency of their regulation using reappraisal.

On the other hand, in a study where two different emotion regulation strategies were compared, reappraisal and suppression, Olatunji, Berg and Zhao *et al*. (2017) found that reappraisal was more effective for disgust compared to fear, while suppression was equally effective in both cases (Olatunji, Berg, & Zhao, 2017). In our study, the magnitude of reduction in emotion rating with reappraisal was similar for both negative emotions, indicating that as a strategy to reduce emotion intensity reappraisal may be equally effective in both negative emotions, albeit with the difference in efficiency discussed above.

Gender differences in emotion processing have previously been reported in numerous studies (Spaapen, Waters, Brummer, Stopa & Bucks, 2014; Tamres, Janicki & Helgeson, 2002) and emotion processing has been claimed to be characterized by sexual dimorphism (Anderson & Adolphs, 2014; Anderson & Rutherford, 2012; Baron‐Cohen, 2004). Indeed, studies linking sex and emotion suggest that gender, hormonal, and reproductive status may influence emotional processes including its induction and regulation aspects (Anderson & Rutherford, 2012; Bradley *et al*., 2001; Conway, Jones, DeBruine *et al*., 2007; Derntl, Windischberger, Robinson *et al*., 2008; Pearson & Lewis, 2005). In the present study, in line with existing literature, we found a significant gender effect during emotion induction, with females reporting roughly 25% higher emotion rating compared to men for both negative emotions, but with no concomitant differences in EDA. It is interesting to note that valence and unpleasantness of a stimulus per se have not been found to differ between the genders (Skolnick, 2013) and that states with high levels of female sex hormones have not been found to be associated with increased anxiety and stress responses as such (Anderson & Rutherford, 2012; Barclay & Barclay, 1976; Pearson *et al*., 2009), suggesting a decoupling of the two phenomena: experienced emotion intensity and hyperarousal (Pearson *et al*., 2009; Swain *et al*., 2007). Our results support this notion. Numerous researchers have also found that women use a wider range of regulation strategies and specifically higher rates of reappraisal compared to men (Spaapen *et al*., 2014; Tamres *et al*., 2002), which was not found to be statistically significant in our study.

### *fNIRS*

Lateral PFC in general, and dorsolateral PFC in particular have repeatedly been shown to be associated with cognitive control of behavior, including that of emotions (Banich *et al*., 2009; Fitzgerald *et al*., 2018; Phan *et al*., 2005; Scult, Knodt, Radtke, Brigidi & Hariri, 2019); therefore, we expected involvement of these areas during the emotion regulation task. Medial PFC has been associated with emotion processing, both in implicit and explicit emotion regulation and in the processing of valence and valuation of emotive stimuli (Fitzgerald *et al*., 2018; Ochsner *et al*., 2002; Phillips *et al*., 2008), why we also expected activation of medial PFC, mainly during emotion induction and possibly also even during emotion regulation.

In our study using fNIRS, we found indeed significant activations in medial PFC for both disgust and fear during emotion induction. During emotion regulation on the other hand, we observed less prefrontal activation, with only activation of the left lateral PFC remaining significant during disgust. This can at first sight be seen as contrasting to the findings of Ochsner *et al*. (2002, 2004) that demonstrated an increased PFC recruitment during regulation of negative emotions. However, one substantial difference is that in these studies, event‐related designs were employed, during which emotion induction and emotion regulation conditions were alternated within seconds, whereas in the present study we used a block design. We suggest that although initial activation of PFC areas is likely to mediate the effortful regulation of emotion and down‐regulation of subcortical areas such as the amygdala, what our design allows for is enough time for successful regulation and to reach a steady state under which emotion intensity is attenuated and under cognitive control, resulting in less need for net activation of prefrontal areas and presumably reflecting decreased activation of the limbic areas that Ochsner *et al*. (2002, 2004) reported. Indeed, studies of emotion regulation on a larger time scale have shown reduction in activation in prefrontal areas after successful emotion regulation treatment (Beauregard, 2014). The fact that medial PFC activations are not seen under conditions of effortful emotion regulation supports this hypothesis and could be demonstrative of a mechanism or an outcome of this process, echoing correspondingly reduced activation of subcortical areas, such as amygdala, with which MPFC is reciprocally interconnected with (Pessoa, 2008; Phan *et al*., 2004). Furthermore, in our study both fNIRS (reflecting PFC activity) and EDA (reflecting peripheral ANS activity), show blunted activation during emotion regulation, a finding which we believe validates our initial hypothesis.

It has been argued (Pessoa, 2008) that valuation of the significance of emotive stimuli and choice of ensuing behavior is impacted both by top‐down frontoparietal attentional systems and bottom‐up emotion modulatory systems in the orbitofrontal PFC and amygdala. In other words, emotion induction per se is likely to essentially entail parallel processes of emotion evaluation and implicit or explicit emotion regulation that would result in the activation of widespread prefrontal areas. It has also been proposed that emotion generation and regulation are two sides of the same coin, engaging the same neural networks (Clark‐Polner, 2016) and experientially differing only in regard to whether one perceives agency over this experience or not (Gross & Barrett, 2011). Our findings showing greater PFC activation during the emotion induction task (representing different affective processes such as detection, identification, evaluation and implicit regulation) compared to the emotion regulation task, where subjects had successfully downregulated the ensuing emotion by roughly 45%, and now not requiring activation to a similar extent of the various affective processes described above, is congruent with the views of those proposing a continuum of processes relevant for both emotion generation and regulation (Clark‐Polner, Johnson & Barrett, 2017; Gross, 2015; Gross & Barrett, 2011; Pessoa, 2008, 2018). As is known, fNIRS has low cortical penetration and does not capture activity in deeper areas like the insula and amygdala, which could have given important information in relation to the PFC activations. The fact that we cannot correlate cortical and subcortical activations using fNIRS imposes limitations on the conclusions that can be drawn from these experiments. For example, Ochsner *et al*. (2002) using fMRI and event‐related design which gives better spatial and temporal resolution than block design with fNIRS (Cui *et al*., 2011; Strangman *et al*., 2002) showed increased PFC activation and decreased amygdala activation during emotion regulation. It should be stated, however, that in the Ochsner *et al*. (2002) study, there were also PFC areas with decreased activation, consistent with our findings here.

#### Differences between fear and disgust

It is generally accepted that fear is elicited by emotive stimuli that involve physical threat (LeDoux, 2014; Mobbs *et al*., 2020) and disgust by sensation of distaste related to threat of disease or contamination (Anderson & Rutherford, 2012; Calder *et al*., 2001; Olatunji, Haidt, McKay & David, 2008). There is also evidence that fear and disgust may differ in their expression (Ekman, 1992), physiology (Ekman *et al*., 1983; Lang *et al*., 1993; Woody & Teachman, 2000) and neural activity they elicit (Phan *et al*., 2004; Phillips *et al*., 1997; Saarimäki, Gotsopoulos, Jääskeläinen *et al*., 2016). We therefore expected that fear and disgust would differentially activate the prefrontal cortex. In our present investigation, we indeed found a number of similarities and differences in the induction and regulation of the two negative emotions, both when compared to baseline conditions and when directly compared to each other.

As previously discussed, both emotions show the same pattern of greater activation of prefrontal areas during emotion induction compared to emotion regulation and both emotions recruited medial prefrontal areas under conditions of emotion induction. However, we found a more widespread PFC activation for disgust, which attained statistical significance in the right (ventrolateral) PFC when directly compared with fear. Right inferior frontal gyrus and right inferior frontal junction have in many studies been implicated in response inhibition (Aron, Robbins & Poldrack, 2004; Bari & Robbins, 2013; Konishi, Nakajima, Uchida, Sekihara & Miyashita, 1998; Swick, Ashley & Turken, 2008), which supported our assumption that a parallel automatic response inhibition process might be going on during disgust to suppress behavior, such as motoric emetic reactions (Skolnick, 2013). We also saw a stronger activation for disgust during emotion regulation in left lateral PFC when compared to baseline conditions, which however did not attain statistical significance when the two negative emotions were directly compared with each other. The above findings suggest greater differences in brain activation patterns during the induction of the two emotions, fear and disgust while the activation patterns converge to no significant differences during conditions of effortful emotion regulation.

### Electrodermal activity

Autonomic responses are considered to have a role in arousal and bodily expression of emotions, conferring social significance to subjective experience of emotion and in this way facilitating interpersonal communication (Critchley, 2002, 2009; Damasio, 1996; 1999; Ekman *et al*., 1983). Although the notion of consistent relationship between an emotion category and a specific set of autonomic nervous system changes has been challenged (Barrett, Adolphs, Marsella, Martinez & Pollak, 2019), we hypothesized that successful emotion regulation of the same labeled, emotive stimuli under the same conditions and within the same context, in a within‐subject design, would cause reliable changes in sympathetic arousal as reflected in EDA (Jackson, Malmstadt, Larson & Davidson, 2000). Previous studies have shown decreased sympathetic activity during cognitive control of emotion, under low to moderate intensity conditions (Gross, 1998, 2015; Kim, Kim & You, 2017; Sheppes & Meiran, 2007; Shiota & Levenson, 2012). Indeed, for both fear and disgust, the frequency of phasic EDA decreased during emotion regulation.

Previous studies have also shown physiological differences in the responses fear and disgust elicit; for example, it has been reported acceleration of heart rate for fear and deceleration for disgust (Cisler *et al*., 2009; Ekman *et al*., 1983; Lang *et al*., 1993) suggesting a distinct EDA fingerprint for the two negative emotions. Based on these previous findings, we expected an increased sympathetic nerve activity during induction of fear and decreased activity during induction of disgust. Indeed, during emotion induction, we found significant differences in the phasic EDA with increased frequency of skin conductance responses for fear compared to disgust, replicating these previous finding. We conclude that experiencing a stimulus as fearful seems to be more readily associated with sympathetic arousal, whereas experience of disgust favors action plans involving parasympathetic activation. Moreover, compared to the baseline conditions, emotion induction during disgust reduced the frequency of phasic EDA, a reduction that continued to be seen also during emotion regulation. For fear, on the other hand, only during emotion regulation was the frequency of phasic EDA reduced compared to baseline conditions.

The results from electrodermal activity together with the results from hemodynamic PFC responses studied with fNIRS suggest that fear and disgust present a different response profile during conditions of emotion induction, whereas during emotion regulation they seem to be less distinguishable from each other.

### CONCLUSIONS AND FUTURE WORK

In the present study, using behavioral measures, measures of PFC hemodynamic responses (fNIRS) and measures of sympathetic autonomic nervous system activity (EDA), we found intriguing similarities and differences in the induction and regulation of fear and disgust.

We found that effortful emotion regulation with reappraisal is equally effective in reducing subjective emotion intensity for fear and disgust and that the state of successful emotion regulation corresponded to less PFC and sympathetic activation compared to emotion induction.

Consistent with our hypothesis, both the fNIRS activation pattern and the electrodermal activity were more distinct and more selective for the particular emotion during emotion induction, highlighting the differences of the two emotions when they were as most intense. These disparate patterns in the hemodynamic response of the PFC could be reflecting adaptive differences in automatic emotion regulation, an integrated and indistinguishable process during emotion induction, prompting different aspects of cognitive control of emotion to be mobilized in response to a fearful stimulus compared to a disgust‐inducing ones. In addition, subjects' sympathetic nervous system was differentially activated, possibly facilitating a particular and appropriate for the respective emotion action plan: for fear, peripheral sympathetic activation (EDA) that would, in an ecological context, facilitate fight or flight and for disgust, parasympathetic activation (EDA) and response/ motor inhibition (fNIRS), possibly leading to avoidance or withdrawal behaviors.

Interestingly, both fear and disgust were reported to be equally successfully regulated under our experimental conditions and we found that the differences in their hemodynamic and electrodermal profile were blunted under conditions of effortful emotion regulation with reappraisal. Moreover, in our study, although we found that cognitive reappraisal was equally effective for fear and disgust, we also found indications that disgust might have some components that make it less responsive to cognitive control.

The differences and similarities that we found in the induction and regulation of fear and disgust indicate that the brain utilizes common as well as specific strategies for affective processes depending on the emotion modality, something also reflected in the periphery as indicated by EDA. Our study also highlights the importance of emotion regulation with reappraisal as target for therapeutic interventions.

The study was approved by the Stockholm County's ethics committee (Dnr 2013/722–32 and 2014/ 436–32). All subjects were given verbal and written information and gave written informed consent through their signature prior to the start of the experiment, in accordance with the Declaration of Helsinki.

All authors discussed the results and contributed to the final manuscript. MM conceived this work, all authors contributed to the design and implementation of the research. MS carried out the experiments. MS and YW performed the computations. MS and MM wrote the manuscript with support from YW.

The study was funded by Stockholms Läns Landsting. The authors have no conflict of interest to declare.

We would like to acknowledge and sincerely thank Erik Boberg, MD for his assistance in data preprocessing and analysis and Måns Thulin, PhD for consultation on the statistical analysis.

## Supporting information


**Supplementary material**. Induction and regulation of the negative emotions fear and disgust: a functional near infrared spectroscopy study.
