# Supplementary material for: Similarities and differences in the induction and regulation of the negative emotions fear and disgust: A functional near infrared spectroscopy study
Source: Scand J Psychol. 2022 May 30;63(6):581–93. doi: 10.1111/sjop.12836 (PMC9796661; doi:10.1111/sjop.12836)
Supplement: Supplementary file 1 — Supplementary material. Induction and regulation of the negative emotions fear and disgust: a functional near infrared spectroscopy study. [file SJOP-63-581-s001.docx]

Supplementary material - Induction and regulation of the negative emotions fear and disgust: a functional near infrared spectroscopy study

## Methods:

## Emotion Induction and Emotion Regulation tasks - Selection of images

The images used in this study, taken from the International Affective Picture System (IAPS) (Lang, Bradley, & Cuthbert, 2008), include SAM (Self-Assessment Manikin) scale for valence, arousal, and dominance. They were subsequently tested during a pilot study, where eight individuals rated them for relevance to the emotions we were interested in studying, fear and disgust.

During the present study, we controlled for valence qualitatively and confirmed it was negative for all subjects (as although fear and disgust are considered negatively valenced emotions, theoretically it is possible that they are pleasant for someone).

Subsequently, in our present study, we used the selected images and we also labelled them as inducing ‘fear’ or ‘disgust’ by explicitly giving instructions explaining that ‘pictures planned to be viewed next were chosen for the purpose of inducing the specific emotion’. We assumed therefore, that even if the participants had different internal models and priors regarding what ‘fear’ and ‘disgust’ mean to them, under these controlled conditions the intersubjectability around these emotional concepts, or as Feldman Barrett (Barrett, 2012) has phrased it, by the virtue of collective intentionality, would uniformly influence the trajectory of emotion experience and regulation.

For one of the primary outcomes, the participants were asked to rate the intensity of the induced (or regulated) feeling or emotion. Emotion Induction rating thus, captured arousal (as a physiological and psychological state, and for the former we used EDA as an objective measure as well). The participants were asked to rate the ‘intensity of the experienced (induced or regulated) emotion in response to the image’.

## List of images

Disgust: “dirty toilet” (IAPS 9301), “feces” (IAPS 9008), “sewage rat eating rotten meat” (IAPS 1280), “infected gangrenous wound” (IAPS 3266) and “masks crawling in a dirty pond” (IAPS 1111).

Fear: “hissing snake” (IAPS 1120), “gaping shark, mouth wide open” (IAPS 1930), “attack pitbull, baring its canines” (IAPS 1300), “spider” (IAPS 1200), and “freshly bleeding hand with severed fingers” (IAPS 3150).

## Results:

## 3.1 Behavioral data

| **EI score** | Emotion Induction score (raw in a scale 1-9) | Primary outcome |
| --- | --- | --- |
| **ER score** | Emotion Regulation score (raw in a scale 1-9) | Primary outcome |
| **ER index** | Emotion Regulation Index = (EI– ER) / EI | Primary outcome |

## 3.1.1 Linear mixed models

**Fixed effects** (interaction):

| Emotion | Fear and Disgust |
| --- | --- |
| Condition | Emotion Induction and Emotion Regulation |

**Random effects**:

Subject effect, due to repeated measures (each subject has data on fear, disgust, during EI and ER)

Wald chi2(3) = 270.57, Prob > chi2 = 0.0000

The intraclass correlation coefficient (ICC1) was moderately high (0.48), indicating that a significant amount of variance could be explained within subjects.

| ***Behavioural data*** | |  |  |  |  |  |
| --- | --- | --- | --- | --- | --- | --- |
| **Main effects** |  |  |  |  |  |  |
| **Predictor** | **Estimate** | **SE** | **z** | **p** | **95% CI (ul)** | **95% CI (ll)** |
| **Emotion** | 0.384 | 0.219 | 1.750 | .080 | -0.046 | 0.813 |
| **Conditon** | -2.482 | 0.216 | -11.487 | <.001 | -2.905 | -2.058 |
| **Interaction** | -0.070 | 0.309 | -0.228 | .820 | -0.676 | 0.535 |
| **Intercept** | 5.451 | 0.214 | 25.527 | <.001 | 5.033 | 5.870 |
|  |  |  |  |  |  |  |
| **Comparisons of interest** | **Contrast** | **SE** | **z** | **p** | **95% CI (ul)** | **95% CI (ll)** |
| **Fear_ER vs Fear_EI** | -2.482 | 0.216 | -11.487 | <.001 | -2.905 | -2.058 |
| **Disgust_EI vs Fear_EI** | 0.384 | 0.219 | 1.750 | .080 | -0.046 | 0.813 |
| **Disgust_ER vs Fear_ER** | 0.313 | 0.219 | 1.429 | .153 | -0.116 | 0.743 |
| **Disgust_ER vs Disgust_EI** | -2.552 | 0.221 | -11.559 | <.001 | -2.985 | -2.119 |

## 3.1.2 T-tests

Paired t-tests, two-sided, 95% CI, Df = 44:

| ***Behavioural data*** | |  |  |  |  |  |
| --- | --- | --- | --- | --- | --- | --- |
| **ER index*** |  |  |  |  |  |  |
|  | **n** | **mean** | **SD** | **p** | **t** | **SE difference** |
| **Fear** | 45 | 0.440 | 0.227 | .593 | 0.538 | 0.017 |
| **Disgust** | 45 | 0.431 | 0.224 |  |  |  |
| * ER index = (EI-ER)/EI |  |  |  |  |  |  |

Independent samples t-tests, 95% CI, two-sided, 95% CI, Df =43:

| ***Behavioural data*** | |  |  |  |  |  |  |
| --- | --- | --- | --- | --- | --- | --- | --- |
| **Gender** | **Females (n=26)** | | **Males (n=19)** | |  |  |  |
|  | **mean** | **SD** | **mean** | **SD** | **p** | **t** | **SE difference** |
| **Fear - EI score** | 6.062 | 1.349 | 4.505 | 1.410 | .001 | -3.750 | 0.415 |
| **Fear - ER index** | 0.477 | 0.206 | 0.394 | 0.244 | .223 | -1.236 | 0.067 |
| **Disgust - EI score** | 6.483 | 1.386 | 4.937 | 1.438 | .001 | -3.575 | 0.433 |
| **Disgust - ER index** | 0.477 | 0.216 | 0.388 | 0.237 | .207 | -1.282 | 0.069 |

## 3.2 Functional near-infrared spectroscopy recordings (fNIRS)

| **Region-wise analysis** | Left (LPFC), Right (RPFC), Medial (MPFC) prefrontal cortex | Primary outcome |
| --- | --- | --- |
| **Channel-wise analysis** | Channels 1-16 | Secondary (exploratory) outcome |

### 3.2.1 Linear mixed models

**Fixed effects** (interaction):

| Emotion | Fear and Disgust |
| --- | --- |
| Condition | Emotion induction and Emotion Regulation |

**Random effects:**

Subject effect, due to repeated measures (each subject has data on fear, disgust, during EI and ER)

#### 3.2.1.1 oxy-Hb

LPFC: Wald chi2(3) = 4.85, Prob > chi2 = 0.1831

RPFC: Wald chi2(3) = 6.26, Prob > chi2 = 0.0997

MPFC: Wald chi2(3) = 6.28, Prob > chi2 = 0.0986

The intraclass correlation coefficients (ICC) for all three areas were 0.18-0.26

| ***RPFC*** |  |  |  |  |  |  |
| --- | --- | --- | --- | --- | --- | --- |
| **Main effects** |  |  |  |  |  |  |
| **Predictor** | **Estimate** | **SE** | **z** | **p** | **95% CI (ul)** | **95% CI (ll)** |
| **Emotion** | 0.306 | 0.186 | 1.641 | .101 | -0.059 | 0.670 |
| **Conditon** | -0.084 | 0.186 | -0.449 | .653 | -0.449 | 0.281 |
| **Interaction** | -0.152 | 0.262 | -0.582 | .560 | -0.666 | 0.361 |
| **Intercept** | 0.255 | 0.150 | 1.703 | .089 | -0.038 | 0.549 |
|  |  |  |  |  |  |  |
| **Comparisons of interest** | **Contrast** | **SE** | **z** | **p** | **95% CI (ul)** | **95% CI (ll)** |
| **Fear_ER vs Fear_EI** | -0.084 | 0.186 | -0.449 | .653 | -0.449 | 0.281 |
| **Disgust_EI vs Fear_EI** | 0.306 | 0.186 | 1.641 | .101 | -0.059 | 0.670 |
| **Disgust_ER vs Fear_ER** | 0.153 | 0.186 | 0.822 | .411 | -0.212 | 0.518 |
| **Disgust_ER vs Disgust_EI** | -0.236 | 0.184 | -1.283 | .199 | -0.597 | 0.124 |
|  |  |  |  |  |  |  |
|  |  |  |  |  |  |  |
| ***MPFC*** |  |  |  |  |  |  |
| **Main effects** |  |  |  |  |  |  |
| **Predictor** | **Estimate** | **SE** | **z** | **p** | **95% CI (ul)** | **95% CI (ll)** |
| **Emotion** | 0.035 | 0.189 | 0.187 | .852 | -0.335 | 0.405 |
| **Conditon** | -0.174 | 0.189 | -0.924 | .355 | -0.544 | 0.195 |
| **Interaction** | -0.238 | 0.265 | -0.899 | .369 | -0.758 | 0.281 |
| **Intercept** | 0.514 | 0.155 | 3.319 | .001 | 0.210 | 0.817 |
|  |  |  |  |  |  |  |
| **Comparisons of interest** | **Contrast** | **SE** | **z** | **p** | **95% CI (ul)** | **95% CI (ll)** |
| **Fear_ER vs Fear_EI** | -0.174 | 0.189 | -0.924 | .355 | -0.544 | 0.195 |
| **Disgust_EI vs Fear_EI** | 0.035 | 0.189 | 0.187 | .852 | -0.335 | 0.405 |
| **Disgust_ER vs Fear_ER** | -0.203 | 0.189 | -1.077 | .282 | -0.573 | 0.167 |
| **Disgust_ER vs Disgust_EI** | -0.413 | 0.186 | -2.214 | .027 | -0.778 | -0.047 |
|  |  |  |  |  |  |  |
|  |  |  |  |  |  |  |
| ***LPFC*** |  |  |  |  |  |  |
| **Main effects** |  |  |  |  |  |  |
| **Predictor** | **Estimate** | **SE** | **z** | **p** | **95% CI (ul)** | **95% CI (ll)** |
| **Emotion** | 0.339 | 0.169 | 2.010 | .044 | 0.009 | 0.670 |
| **Conditon** | -0.023 | 0.169 | -0.138 | .891 | -0.354 | 0.308 |
| **Interaction** | -0.316 | 0.238 | -1.332 | .183 | -0.782 | 0.149 |
| **Intercept** | 0.116 | 0.132 | 0.880 | .379 | -0.143 | 0.375 |
|  |  |  |  |  |  |  |
| **Comparisons of interest** | **Contrast** | **SE** | **z** | **p** | **95% CI (ul)** | **95% CI (ll)** |
| **Fear_ER vs Fear_EI** | -0.023 | 0.169 | -0.138 | .891 | -0.354 | 0.308 |
| **Disgust_EI vs Fear_EI** | 0.339 | 0.169 | 2.010 | .044 | 0.009 | 0.670 |
| **Disgust_ER vs Fear_ER** | 0.023 | 0.169 | 0.136 | .892 | -0.308 | 0.354 |
| **Disgust_ER vs Disgust_EI** | -0.340 | 0.167 | -2.034 | .042 | -0.667 | -0.012 |

#### 3.2.1.2 deoxy-Hb

LPFC: Wald chi2(3) = 2.33, Prob > chi2 = 0.5065

MPFC: Wald chi2(3) =6.47, Prob > chi2 = 0.0908

RPFC: Wald chi2(3) =2.76, Prob > chi2 = 0.4299

The intraclass correlation coefficients (ICC) for all three areas were 0.19-0.36

| ***RPFC*** |  |  |  |  |  |  |
| --- | --- | --- | --- | --- | --- | --- |
| **Main effects** |  |  |  |  |  |  |
| **Predictor** | **Estimate** | **SE** | **z** | **p** | **95% CI (ul)** | **95% CI (ll)** |
| **Emotion** | 0.096 | 0.060 | 1.599 | .110 | -0.022 | 0.214 |
| **Conditon** | 0.045 | 0.060 | 0.757 | .449 | -0.072 | 0.163 |
| **Interaction** | -0.071 | 0.084 | -0.841 | .400 | -0.237 | 0.095 |
| **Intercept** | -0.026 | 0.053 | -0.496 | .620 | -0.129 | 0.077 |
|  |  |  |  |  |  |  |
| **Comparisons of interest** | **Contrast** | **SE** | **z** | **p** | **95% CI (ul)** | **95% CI (ll)** |
| **Fear_ER vs Fear_EI** | 0.045 | 0.060 | 0.757 | .449 | -0.072 | 0.163 |
| **Disgust_EI vs Fear_EI** | 0.096 | 0.060 | 1.599 | .110 | -0.022 | 0.214 |
| **Disgust_ER vs Fear_ER** | 0.025 | 0.060 | 0.418 | .676 | -0.093 | 0.143 |
| **Disgust_ER vs Disgust_EI** | -0.026 | 0.059 | -0.431 | .667 | -0.142 | 0.091 |
|  |  |  |  |  |  |  |
|  |  |  |  |  |  |  |
| ***MPFC*** |  |  |  |  |  |  |
| **Main effects** |  |  |  |  |  |  |
| **Predictor** | **Estimate** | **SE** | **z** | **p** | **95% CI (ul)** | **95% CI (ll)** |
| **Emotion** | -0.101 | 0.075 | -1.334 | .182 | -0.249 | 0.047 |
| **Conditon** | 0.028 | 0.076 | 0.373 | .709 | -0.120 | 0.176 |
| **Interaction** | -0.064 | 0.106 | -0.599 | .549 | -0.272 | 0.145 |
| **Intercept** | 0.072 | 0.059 | 1.223 | .221 | -0.044 | 0.188 |
|  |  |  |  |  |  |  |
| **Comparisons of interest** | **Contrast** | **SE** | **z** | **p** | **95% CI (ul)** | **95% CI (ll)** |
| **Fear_ER vs Fear_EI** | 0.028 | 0.076 | 0.373 | .709 | -0.120 | 0.176 |
| **Disgust_EI vs Fear_EI** | -0.101 | 0.075 | -1.334 | .182 | -0.249 | 0.047 |
| **Disgust_ER vs Fear_ER** | -0.164 | 0.075 | -2.177 | .029 | -0.312 | -0.016 |
| **Disgust_ER vs Disgust_EI** | -0.035 | 0.075 | -0.474 | .635 | -0.182 | 0.111 |
|  |  |  |  |  |  |  |
|  |  |  |  |  |  |  |
| ***LPFC*** |  |  |  |  |  |  |
| **Main effects** |  |  |  |  |  |  |
| **Predictor** | **Estimate** | **SE** | **z** | **p** | **95% CI (ul)** | **95% CI (ll)** |
| **Emotion** | -0.063 | 0.049 | -1.279 | .201 | -0.158 | 0.033 |
| **Conditon** | 0.001 | 0.049 | 0.025 | .980 | -0.095 | 0.097 |
| **Interaction** | 0.053 | 0.069 | 0.770 | .441 | -0.082 | 0.188 |
| **Intercept** | 0.048 | 0.041 | 1.177 | .239 | -0.032 | 0.128 |
|  |  |  |  |  |  |  |
| **Comparisons of interest** | **Contrast** | **SE** | **z** | **p** | **95% CI (ul)** | **95% CI (ll)** |
| **Fear_ER vs Fear_EI** | 0.001 | 0.049 | 0.025 | .980 | -0.095 | 0.097 |
| **Disgust_EI vs Fear_EI** | -0.063 | 0.049 | -1.279 | .201 | -0.158 | 0.033 |
| **Disgust_ER vs Fear_ER** | -0.010 | 0.049 | -0.197 | .844 | -0.106 | 0.086 |
| **Disgust_ER vs Disgust_EI** | 0.054 | 0.048 | 1.121 | .262 | -0.041 | 0.149 |

### 3.2.2 T-tests (oxy-Hb)

#### fNIRS, Fear – EI (Emotion induction task contrast Rest)

one-sample t-tests, two-sided, 95% CI, Df =41. n=42

| ***PFC activations during Emotion Induction, Fear*** | | | |  |  |
| --- | --- | --- | --- | --- | --- |
| **fNIRS Channel** | **mean** | **SD** | **SE** | **p** | **t** |
| 1 | 0.402 | 1.082 | 0.167 | .021 | 2.409 |
| 2 | 0.226 | 1.015 | 0.157 | .157 | 1.442 |
| 3 | 0.050 | 1.113 | 0.172 | .771 | 0.293 |
| 4 | 0.112 | 1.172 | 0.181 | .539 | 0.620 |
| 5 | 0.272 | 1.074 | 0.166 | .108 | 1.643 |
| 6 | 0.307 | 1.090 | 0.168 | .075 | 1.826 |
| 7 | 0.361 | 1.223 | 0.189 | .062 | 1.916 |
| 8 | 0.581 | 1.440 | 0.222 | .012 | 2.617 |
| 9 | 0.387 | 1.059 | 0.163 | .023 | 2.365 |
| 10 | 0.710 | 1.553 | 0.240 | .005 | 2.962 |
| 11 | 0.101 | 0.840 | 0.130 | .442 | 0.776 |
| 12 | 0.089 | 0.942 | 0.145 | .542 | 0.614 |
| 13 | 0.078 | 0.875 | 0.135 | .565 | 0.580 |
| 14 | -0.086 | 0.880 | 0.136 | .530 | -0.634 |
| 15 | 0.299 | 0.932 | 0.144 | .044 | 2.078 |
| 16 | 0.109 | 0.771 | 0.119 | .363 | 0.920 |

#### fNIRS, Disgust - EI (Emotion induction task contrast Rest)

#### one-sample t-tests, two-sided, 95% CI, Df =42. n=43

| ***PFC activations during Emotion Induction, Disgust*** | | | |  |  |
| --- | --- | --- | --- | --- | --- |
| **fNIRS Channel** | **mean** | **SD** | **SE** | **p** | **t** |
| 1 | 0.662 | 0.979 | 0.149 | < .001 | 4.434 |
| 2 | 0.606 | 1.264 | 0.193 | .003 | 3.142 |
| 3 | 0.512 | 1.066 | 0.163 | .003 | 3.150 |
| 4 | 0.415 | 1.133 | 0.173 | .021 | 2.404 |
| 5 | 0.560 | 0.923 | 0.141 | < .001 | 3.981 |
| 6 | 0.528 | 1.327 | 0.202 | .012 | 2.612 |
| 7 | 0.551 | 0.990 | 0.151 | .001 | 3.650 |
| 8 | 0.593 | 1.498 | 0.228 | .013 | 2.594 |
| 9 | 0.651 | 0.926 | 0.141 | < .001 | 4.606 |
| 10 | 0.535 | 1.077 | 0.164 | .002 | 3.255 |
| 11 | 0.505 | 0.799 | 0.122 | < .001 | 4.149 |
| 12 | 0.542 | 1.111 | 0.169 | .003 | 3.198 |
| 13 | 0.347 | 0.778 | 0.119 | .006 | 2.926 |
| 14 | 0.539 | 1.021 | 0.156 | .001 | 3.462 |
| 15 | 0.452 | 0.969 | 0.148 | .004 | 3.061 |
| 16 | 0.382 | 1.374 | 0.210 | .076 | 1.822 |

#### fNIRS, Fear – ER (Emotion regulation task contrast Rest)

one-sample t-tests, two-sided, 95% CI, Df =41. n=42

| ***PFC activations during Emotion Regulation, Fear*** | | | |  |  |
| --- | --- | --- | --- | --- | --- |
| **fNIRS Channel** | **mean** | **SD** | **SE** | **p** | **t** |
| 1 | 0.198 | 1.146 | 0.177 | .269 | 1.121 |
| 2 | 0.233 | 1.340 | 0.207 | .266 | 1.127 |
| 3 | 0.017 | 1.033 | 0.159 | .916 | 0.106 |
| 4 | 0.057 | 1.154 | 0.178 | .749 | 0.322 |
| 5 | 0.247 | 1.021 | 0.157 | .124 | 1.570 |
| 6 | 0.272 | 1.251 | 0.193 | .167 | 1.407 |
| 7 | 0.166 | 1.226 | 0.189 | .387 | 0.875 |
| 8 | 0.359 | 1.397 | 0.216 | .104 | 1.665 |
| 9 | 0.221 | 0.963 | 0.149 | .145 | 1.487 |
| 10 | 0.404 | 1.306 | 0.201 | .051 | 2.008 |
| 11 | 0.095 | 1.048 | 0.162 | .560 | 0.587 |
| 12 | 0.107 | 1.277 | 0.197 | .589 | 0.545 |
| 13 | -0.013 | 0.988 | 0.152 | .930 | -0.088 |
| 14 | -0.064 | 1.181 | 0.182 | .727 | -0.352 |
| 15 | 0.182 | 0.966 | 0.149 | .228 | 1.224 |
| 16 | 0.228 | 1.217 | 0.188 | .232 | 1.213 |

#### fNIRS, Disgust – ER (Emotion regulation task contrast Rest)

one-sample t-tests, two-sided, 95% CI, Df =42. n=43

| **PFC activations during Emotion Regulation. Disgust** | | | |  |  |
| --- | --- | --- | --- | --- | --- |
| **fNIRS Channel** | **mean** | **SD** | **SE** | **p** | **t** |
| 1 | 0.352 | 0.813 | 0.124 | .007 | 2.843 |
| 2 | 0.306 | 0.858 | 0.131 | .024 | 2.334 |
| 3 | 0.320 | 0.835 | 0.127 | .016 | 2.513 |
| 4 | 0.244 | 0.941 | 0.143 | .096 | 1.701 |
| 5 | 0.161 | 0.770 | 0.117 | .177 | 1.374 |
| 6 | 0.278 | 1.061 | 0.162 | .094 | 1.716 |
| 7 | 0.228 | 0.869 | 0.132 | .092 | 1.723 |
| 8 | 0.163 | 0.893 | 0.136 | .238 | 1.197 |
| 9 | 0.261 | 0.696 | 0.106 | .018 | 2.463 |
| 10 | 0.075 | 1.009 | 0.154 | .628 | 0.489 |
| 11 | 0.085 | 0.927 | 0.141 | .550 | 0.603 |
| 12 | 0.081 | 0.822 | 0.125 | .523 | 0.645 |
| 13 | 0.130 | 0.772 | 0.118 | .276 | 1.104 |
| 14 | 0.124 | 0.806 | 0.123 | .319 | 1.009 |
| 15 | 0.218 | 0.803 | 0.122 | .082 | 1.782 |
| 16 | 0.109 | 0.848 | 0.129 | .404 | 0.842 |

#### fNIRS, Fear versus Disgust - Emotion Induction task contrast Rest

paired t-tests, two-sided, 95% CI, Df =39, n=40

| **fNIRS Channel** | **Mean Disgust** | **SD Disgust** | **Mean Fear** | **SD Fear** | **p** | **t** | **SE difference** |
| --- | --- | --- | --- | --- | --- | --- | --- |
| 1 | 0.800 | 1.385 | 0.544 | 1.519 | .183 | -1.354 | 0.189 |
| 2 | 0.612 | 1.531 | 0.378 | 1.853 | .476 | -0.719 | 0.325 |
| 3 | 0.590 | 1.559 | -0.233 | 2.580 | .113 | -1.620 | 0.508 |
| 4 | 0.397 | 1.224 | 0.147 | 1.552 | .357 | -0.932 | 0.268 |
| 5 | 0.568 | 1.031 | 0.207 | 2.016 | .313 | -1.021 | 0.354 |
| 6 | 0.486 | 1.587 | 0.299 | 1.175 | .540 | -0.618 | 0.301 |
| 7 | 0.540 | 1.125 | 0.321 | 1.993 | .548 | -0.606 | 0.361 |
| 8 | 0.511 | 2.127 | 0.715 | 2.621 | .689 | 0.403 | 0.508 |
| 9 | 0.684 | 1.007 | 0.325 | 1.703 | .224 | -1.235 | 0.291 |
| 10 | 0.348 | 1.600 | 0.889 | 2.358 | .159 | 1.435 | 0.377 |
| 11 | 0.560 | 0.896 | -0.068 | 1.683 | .051 | -2.018 | 0.311 |
| 12 | 0.549 | 1.337 | -0.107 | 2.035 | .076 | -1.822 | 0.360 |
| 13 | 0.478 | 1.174 | 0.066 | 1.096 | .075 | -1.829 | 0.225 |
| 14 | 0.644 | 1.220 | -0.121 | 1.130 | .011 | -2.678 | 0.286 |
| 15 | 0.554 | 1.217 | 0.478 | 1.457 | .680 | -0.416 | 0.182 |
| 16 | 0.419 | 1.512 | 0.482 | 2.293 | .843 | 0.200 | 0.320 |

## 3.3 Electrodermal activity (EDA)

| **Frequency EDR** | the frequency of phasic skin conductance response (SCR) | Primary outcome |
| --- | --- | --- |
| **Mean EDR amplitude** | the magnitude, ie mean amplitude, of the phasic skin conductance response (SCR) | Secondary outcome |
| **Mean EDL** | the mean amplitude of the tonic electrodermal activity level | Secondary outcome |

| **EI** | electrodermal responses during Emotion Induction |
| --- | --- |
| **ER** | electrodermal responses during Emotion Regulation |
| **Rest** | nonspecific electrodermal responses |

### 3.3.1 Linear mixed models (EDA frequency)

**Fixed effects** (interaction):

| Emotion | Fear and Disgust |
| --- | --- |
| Condition | Emotion induction and Emotion Regulation |

**Random effects**:

Subject effect, due to repeated measures (each subject has data on fear, disgust, during EI and ER)

Wald chi2(3) = 447.46, Prob > chi2 = 0.0000

The intraclass correlation coefficient (ICC) was low (0.007).

| ***EDA frequency*** | |  |  |  |  |  |
| --- | --- | --- | --- | --- | --- | --- |
| **Main effects** |  |  |  |  |  |  |
| **Predictor** | **Estimate** | **SE** | **z** | **p** | **95% CI (ul)** | **95% CI (ll)** |
| **Emotion** | -0.015 | 0.007 | -2.308 | .021 | -0.028 | -0.002 |
| **Conditon** | 0.087 | 0.007 | 13.024 | <.001 | 0.074 | 0.100 |
| **Interaction** | 0.024 | 0.009 | 2.566 | .010 | 0.006 | 0.043 |
| **Intercept** | -0.001 | 0.005 | -0.192 | .847 | -0.010 | 0.008 |
|  |  |  |  |  |  |  |
| **Comparisons of interest** | **Contrast** | **SE** | **z** | **p** | **95% CI (ul)** | **95% CI (ll)** |
| **Fear_ER vs Fear_EI** | 0.087 | 0.007 | 13.024 | <.001 | 0.074 | 0.100 |
| **Disgust_EI vs Fear_EI** | -0.015 | 0.007 | -2.308 | .021 | -0.028 | -0.002 |
| **Disgust_ER vs Fear_ER** | 0.009 | 0.007 | 1.322 | .186 | -0.004 | 0.022 |
| **Disgust_ER vs Disgust_EI** | 0.111 | 0.007 | 16.654 | <.001 | 0.098 | 0.124 |

### 3.3.2 T-tests

#### EDA, Fear: EI contrast Rest

paired t-tests, two-sided, 95% CI, Df =38, n=39

| ***EDA fear*** |  |  |  |  |  |  |  |
| --- | --- | --- | --- | --- | --- | --- | --- |
|  | **Rest** | | **EI** | |  |  |  |
|  | **mean** | **SD** | **mean** | **SD** | **p** | **t** | **SE difference** |
| **Frequency EDR (EDR/time)** | 0.080 | 0.056 | 0.078 | 0.061 | .834 | -0.211 | 0.005 |
| **Mean EDR amplitude** | 0.133 | 0.133 | 0.091 | 0.096 | .062 | -1.925 | 0.022 |
| **Mean EDL** | 5.929 | 3.270 | 5.861 | 3.298 | .317 | -1.013 | 0.068 |

#### EDA, Disgust: EI contrast Rest

paired t-tests, two-sided, 95% CI, Df =42. n=43

| ***EDA disgust*** |  |  |  |  |  |  |  |
| --- | --- | --- | --- | --- | --- | --- | --- |
|  | **Rest** | | **EI** | |  |  |  |
|  | **mean** | **SD** | **mean** | **SD** | **p** | **t** | **SE difference** |
| **Frequency EDR (EDR/time)** | 0.074 | 0.057 | 0.056 | 0.052 | <.001 | -4.378 | 0.004 |
| **Mean EDR amplitude** | 0.121 | 0.131 | 0.070 | 0.120 | .013 | -2.586 | 0.019 |
| **Mean EDL** | 5.698 | 3.476 | 5.610 | 3.450 | .052 | -1.998 | 0.044 |

#### EDA, Fear: ER contrast Rest

paired t-tests, two-sided, 95% CI, Df =38, n=39

| ***EDA fear*** |  |  |  |  |  |  |  |
| --- | --- | --- | --- | --- | --- | --- | --- |
|  | **Rest** | | **ER** | |  |  |  |
|  | **mean** | **SD** | **mean** | **SD** | **p** | **t** | **SE difference** |
| **Frequency EDR (EDR/time)** | 0.080 | 0.056 | 0.073 | 0.054 | .114 | -1.618 | 0.004 |
| **Mean EDR amplitude** | 0.133 | 0.133 | 0.098 | 0.121 | .008 | -2.779 | 0.013 |
| **Mean EDL** | 5.929 | 3.270 | 5.935 | 3.271 | .919 | 0.103 | 0.051 |

#### EDA, Disgust: ER contrast Rest

paired t-tests, two-sided, 95% CI, Df =42. n=43

| ***EDA disgust*** |  |  |  |  |  |  |  |
| --- | --- | --- | --- | --- | --- | --- | --- |
|  | **Rest** | | **ER** | |  |  |  |
|  | **mean** | **SD** | **mean** | **SD** | **p** | **t** | **SE difference** |
| **Frequency EDR (EDR/time)** | 0.074 | 0.057 | 0.056 | 0.050 | <.001 | -4.257 | 0.004 |
| **Mean EDR amplitude** | 0.121 | 0.131 | 0.080 | 0.135 | .009 | -2.725 | 0.015 |
| **Mean EDL** | 5.698 | 3.476 | 5.649 | 3.436 | .198 | -1.307 | 0.037 |

The p-values presented are raw (unadjusted) for multiple comparisons

# Power analysis

All calculations were performed using Stata 14 software (StataCorp. 2015. Stata Statistical Software: Release 14. College Station, TX: StataCorp LP).

## Behaviour data

Judging from a previous study and a pilot study we considered an effect size of at least 0.5 points difference in the scores of subjective experience (scale ranging from 1 to 9). The study would require a sample size of 34 to achieve a power of 80% and a level of significance of 5% (two sided), for detecting an effect size of 0.5 between pairs.

Post-hoc power analysis indicated that our study had a 90% chance of detecting a large effect size (1.5 difference in scores) between genders as significant at the 5% level (two tailed).

## fNIRS

We calculated that our sample size was sufficiently powered (87%) to detect meaningful differences in fNIRS contrasts of different regions (medium effect size 0.5).

Post-hoc power analysis indicated that our study was underpowered (ca 50%) of detecting differences of 0.50 effect size between genders as significant at the 5% level (two tailed), we therefore chose not to perform this analysis.

## EDA

We calculated that our sample size was sufficiently powered (90%) to detect meaningful differences in EDA measures between conditions (differences corresponding to medium effect size 0.50).

Post-hoc power analysis indicated that our study had an 80% chance of detecting difference of a 0.50 effect size between genders as significant at the 5% level (two tailed).

# References

Barrett, L. F. (2012). Emotions Are Real. *Emotion, 12*(3), 413-429. doi:10.1037/a0027555

Lang, P. J., Bradley, M. M., & Cuthbert, B. N. (2008). International affective picture system (IAPS): affective ratings of pictures and instruction manual. *Technical Report A-8, University of Florida, Gainesville, FL.*
